# A Systematic Review and Meta-Analysis of Therapeutic Hypothermia and Pharmacological Cotherapies in Animal Models of Ischemic Stroke

**DOI:** 10.1089/ther.2024.0012

**Published:** 2024-12-16

**Authors:** Angely Claire C. Suerte, Lane J. Liddle, Ashley Abrahart, Elmira Khiabani, Frederick Colbourne

**Affiliations:** ^1^ Department of Psychology, Faculty of Science, University of Alberta, Edmonton, Canada.; ^2^ Neuroscience and Mental Health Institute, University of Alberta, Edmonton, Canada.

**Keywords:** ischemic stroke, therapeutic hypothermia, combination therapies, pharmacological agents, meta-analysis, translational research

## Abstract

Therapeutic hypothermia (TH) lessens ischemic brain injury. Cytoprotective agents can augment protection, although it is unclear which combinations are most effective. The objective of this study is to identify which cytoprotective drug works best with delayed TH. Following PRISMA guidelines, a systematic review (PubMed, Web of Science, MEDLINE, Scopus) identified controlled experiments that used an *in vivo* focal ischemic stroke model and evaluated the efficacy of TH (delay of ≥1 hour) coupled with cytoprotective agents. This combination was our main intervention compared with single treatments with TH, drug, or no treatment. Endpoints were brain injury and neurological impairment. The CAMARADES checklist for study quality and the SYRCLE’s risk of bias tool gauged study quality. Twenty-five studies were included. Most used young, healthy male rats, with only one using spontaneously hypertensive rats. Two studies used mice models, and six used adult animals. Study quality was moderate (median score = 6), and risk of bias was high. Pharmacological agents provided an additive effect on TH for all outcomes measured. Magnesium coupled with TH had the greatest impact compared with other agent-TH combinations on all outcomes. Longer TH durations improved both behavioral and histological outcomes and had greater cytoprotective efficacy than shorter durations. Anti-inflammatories were the most effective in reducing infarction (standardized mean difference [SMD]: −1.64, confidence interval [CI]: [−2.13, −1.15]), sulfonylureas reduced edema the most (SMD: −2.32, CI: [−3.09, −1.54]), and antiapoptotic agents improved behavioral outcomes the most (normalized mean difference: 52.38, CI: [45.29, 59.46]). Statistically significant heterogeneity was observed (*I*^2^ = 82 − 98%, all *p* < 0.001), indicating that studies wildly differ in their effect size estimates. Our results support the superiority of adding cytoprotective therapies with TH (vs. individual or no therapy). Additional exploratory and confirmatory studies are required to identify and thoroughly assess combination therapies owing to limited work and inconsistent translational quality.

## Introduction

Stroke is one of the leading causes of death and disability around the world (Alharbi et al., 2019; Feigin et al., 2017). The most common subtype is ischemic stroke, accounting for over 62% of all strokes annually (Feigin et al., 2022). Only a limited number of treatment options are available for ischemic stroke, such as mechanical thrombectomy (MT) and thrombolysis (Powers et al., 2019). However, not all patients benefit from existing treatments, and many are ineligible. Additional cytoprotective therapies are needed for patients who are ineligible for current treatments and to augment reperfusion therapies. There is considerable evidence to show that delayed therapeutic hypothermia (TH) persistently reduces ischemic brain damage, including both global and focal injuries, which has been frequently reviewed with respect to efficacy and mechanisms of action (Binda et al., 2024; Liddle et al., 2021). There have been some translational successes in adult cardiac arrest (Sagalyn et al., 2009; Scirica, 2013) and hypoxic–ischemic injury (HIE) in newborns, where the evidence for benefit is stronger (Aker et al., 2020; Catherine et al., 2021). For example, several HIE clinical trials have shown that mild TH reduces death and disability, with benefits persisting into childhood (Higgins et al., 2011b). Cardiac patients treated with mild TH also demonstrated improved neurological outcomes and a lower mortality rate over six months (Testori et al., 2011), although recent reviews indicate mixed results with moderate levels of bias in the included studies (Colls Garrido et al., 2021). Furthermore, there is continuing debate about optimal parameters and the extent of benefit of TH in cardiac arrest. Clinical trials for ischemic stroke have also been inconclusive (Kuczynski et al., 2020), perhaps owing to suboptimal treatment parameters (e.g., late intervention times and inadequate dosage), complications (e.g., pneumonia), and the difficulty with finding treatment effects in studies with small sample sizes. For instance, a meta-analysis did not demonstrate neuroprotective efficacy of TH in ischemic stroke patients but did reveal an increased risk of complications (Kuczynski et al., 2020). Similarly, two major TH clinical studies for ischemic stroke (EuroHYPO-1 and ICTuS) were unsuccessful because of high complications and difficulty achieving target temperatures (Lyden et al., 2016; Van Der Worp et al., 2014). These clinical results are a stark contrast to preclinical data, in which multiple meta-analyses have provided demonstrable benefits on both behavioral and histological endpoints (Dumitrascu et al., 2016; Van Der Worp et al., 2007). Despite all these data, however, there are still gaps in our translational knowledge—for example, the optimal TH duration is still unknown, and experimental models are not representative of the clinical condition (Eberle et al., 2023; Liddle et al., 2022). Furthermore, bias reduction is not unanimous, and time-to-treat is short in animals and long in patients, demonstrating disconnect between the two (Liddle et al., 2022).

Although cooling appears to work through many mechanisms, there are limitations that, if further addressed with cotherapies, could augment benefit. We can broadly categorize pharmacological cotherapies as follows: (1) directly inducing TH, (2) aiding cooling, (3) countering other unwanted side effects of cooling (e.g., reducing risk of pneumonia), and (4) acting in an additive or synergistic manner to augment cytoprotection directly or indirectly. The fourth point would include thrombectomy, as well as the use of drugs that have intrinsic cytoprotective properties. Cotherapies may be applied in a staged manner or concomitantly. Use of cotherapies seems to be well established in animal literature (Zhang et al., 2018). For instance, combining TH with antioxidants (e.g., Edaravone) significantly reduces both edema and infarct volume compared with the monotherapies (Nito et al., 2003). Similarly, TH combined with anti-inflammatory agents such as FK506 (i.e., tacrolimus) can also prolong the therapeutic time window of both treatments and further decrease infarct volume compared with the monotherapies (O’Collins et al., 2012). The broad categorization of pharmacological agents and their effects with TH allows us to consider all categories to determine how to effectively increase the efficiency of TH using cotherapies.

Meta-analyses synthesize data from multiple experiments on the same topic, which may allow for a more accurate estimation of efficacy (O’Collins et al., 2012). In addition, methodological and translational rigor may be evaluated with meta-analysis. This is vital, as biased outcome assessments can lead to issues, such as inflated effect sizes and hindered translation efforts (Ioannidis, 2011). Therefore, effectively translating therapeutic interventions into clinical settings is ultimately dependent on high-quality preclinical designs and reporting. Several meta-analyses on TH have already aided our understanding of the quality of studies and provide an accurate understanding of what remains to be elucidated before further clinical work (Dumitrascu et al., 2016; Van Der Worp et al., 2007). Although many studies have directly investigated TH cotherapies, no meta-analyses have formally evaluated the efficacy of TH cotherapies in ischemic stroke. Through a systematic review and meta-analysis, we identified the literature surrounding combination therapy with TH, determined the overall efficacy of cotherapies, and analyzed the translational quality of the studies. We expect study qualities to be generally low and that synergistic or additive effects might depend on each endpoint.

## Methods

### Search terms

We systematically searched the PubMed, MEDLINE, Scopus, and Web of Science databases for English-language articles with no constraints on the date of publication. Our search was complete as of September 19, 2023. The search criteria were created with the following terms:

(((combination therapy OR co-therapy OR mediated OR induc* OR combination OR combined OR combination treatment) AND (therapeutic hypothermia OR targeted temperature management OR cooling OR hypothermia)) AND (ischemic stroke OR ischemic injury OR middle cerebral artery occlusion OR MCAO OR ischemi* OR ischaemia) AND (animal OR rodent OR mouse OR rat OR monkey) NOT (hypoxic ischemic encephalopathy) NOT (cardiac[Title/Abstract]) NOT (spinal cord[Title/Abstract]))) NOT (renal[Title/Abstract]).

The inclusion criteria included only *in vivo* experiments using focal ischemic stroke models (Table 1). Limiting our treatments to delayed therapies (TH delay of ≥ 1 hour after stroke onset and agents administered any time postischemia) more closely mimics clinical scenarios, as patients are not typically treated before or immediately following their stroke onset (Krieger and Yenari, 2004). The study was preregistered (CRD42022341081) with the International Prospective Register of Systematic Reviews (PROSPERO) and was conducted according to the PICOS (Problem, Intervention, Comparison, Outcome, Study Design) framework for systematic reviews. We used Covidence software (Veritas Health Innovation, Melbourne, Australia) for abstract and full-text screening. Three reviewers (A.C.C.S., A.A., and E.K.) independently performed article and full-text screening, as well as data extraction. A fourth reviewer (L.J.L.) served as a tiebreaker when discrepancies occurred. Experimental parameters, descriptive statistics, and treatment parameters for each outcome were collected and extracted into a spreadsheet. Cohen’s kappa was used to quantify inter-rater reliability.

**Table 1.** Inclusion and Exclusion Criteria as Decided *A Priori* (PROSPERO Registration Number: CRD42022341081)

Screening	Include	Exclude
Abstract	1. Stroke type is focal ischemia 2. Study type is animal 3. Combination therapy with TH	1. Stroke type is not focal ischemia 2. Not an animal study 3. Not a combination therapy with TH *If unclear from abstract, study continued to full text review
Full Text	1. Stroke type is focal ischemia 2. Study type is animal 3. TH administration of ≥1 hour after stroke onset 4. Agent administered after stroke onset 5. TH-only group present 6. Full text available in English	1. Stroke type is not focal ischemia 2. Not an animal study 3. TH administered before, during, or <1 hour after stroke onset 4. Agent administered before stroke onset 5. No TH-only group included in the experiments 6. Full text unavailable in English

TH, therapeutic hypothermia.

### Study quality and certainty assessment

We used the SYRCLE risk of bias (SRoB) tool and the CAMARADES checklist for study quality (Hooijmans et al, 2014; Macleod et al, 2004). SRoB was based on the Cochrane Collaboration risk of bias tool and was adapted to examine the biases that arise in animal experiments (Higgins et al., 2011a; Macleod et al., 2004). Study quality was analyzed using the CAMARADES checklist, and each study was given a score out of 10 (Hooijmans et al., 2014). Following the PRISMA 2020 checklist, certainty assessment was also conducted through the GRADE approach, a method described in the Cochrane handbook (Higgins et al., 2020; Page et al., 2021). Measures of certainty, including publication bias, study limitations, and consistency of findings across studies, were assessed (Hooijmans et al., 2018; Page et al., 2021).

### Statistical analysis

Data were analyzed using the statistical software R (version 2022.12.0 + 353, Vienna, Austria). A random effects meta-analysis model was chosen for each endpoint because of anticipated variability in the study designs. Effect sizes were calculated using normalized mean differences (NMDs) for behavior and standardized mean differences (SMDs) for edema and infarct volume. If behavior was assessed multiple times, we combined late assessments (≥14 days after stroke onset) and early assessments (<14 days after stroke onset). Data were extracted according to provided summary statistics (e.g., mean, standard deviation, standard error of the mean) or directly extracted from graphs if not stated. Heterogeneity was assessed using the *I*^2^ statistic (Higgins et al., 2020). Egger regression and trim-and-fill funnel plots were used to determine publication bias. It was decided *a priori* that sensitivity analyses would be conducted when studies disproportionally contributed to the model (e.g., if they had effects that were three times larger than the overall meta-analytic effect) (Riley et al., 2004). Experimental groups were organized into four categories as follows: TH+DRUG, TH, DRUG, and NORMO (normothermic control). Subgroup analyses were conducted on the TH versus NORMO and DRUG versus NORMO comparison groups to explore the impact of TH duration and agent. Meta-regression was conducted on the TH+DRUG versus NORMO comparison to determine which agent categories affected outcomes when combined with TH. To see if the effects of the pharmacological agents on TH were additive (i.e., combination treatment efficacy equals the sum of the individual therapies) or synergistic (i.e., combination treatment efficacy is greater than the sum of the individual therapies), we compared the confidence intervals (CI) of the combination treatment versus agent comparison with the TH versus control comparison (O’Collins et al., 2012). CI overlapping between the two groups indicated additive effects (e.g., µ_Combo_ = µ_Agent_ + µ_TH_ and therefore µ_Combo_ − µ_TH_ = µ_Agent_).

## Results

### Search results

**FIG. 1.**
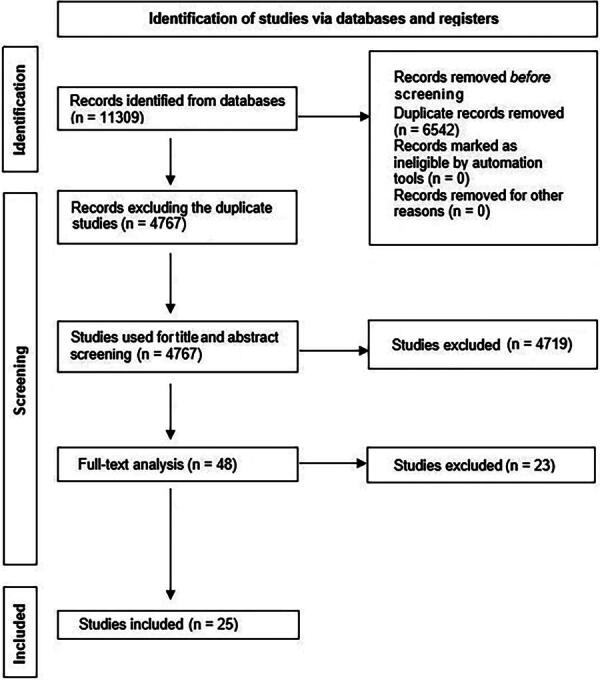
PRISMA diagram of the number of studies returned by our database searches as of September 19, 2023.

We identified 4767 articles in our search (Fig. 1). Of these, 4719 abstracts were omitted for being irrelevant (e.g., nonstroke, nonexperimental, noncombination therapy studies), and 23 full studies were excluded after full-text screening—17 for improper TH and/or agent parameters (e.g., administering treatments before and/or <1 hour after stroke onset), 2 for not using a pharmacological intervention, 2 for not having the proper control group (TH-only group), 1 for not using consistent dosing parameters between groups, and 1 for administering TH > 35°C. Therefore, we included 25 studies in the final analysis (An et al., 2017; Aronowski et al., 2003; Cai et al., 2017, 2016a, 2016b; Campbell et al., 2013, 2008; Cechmanek et al., 2015; Dai et al., 2021; Ghahari et al., 2014; Hassanipour et al., 2020; Kallmünzer et al., 2012; Kollmar et al., 2004; Lee et al., 2016; Meloni et al., 2013; Song et al., 2013; Tang et al., 2013; Wang et al., 2003, 2002, 2012; Wu et al., 2019, 2016; Yu et al., 2020; Zhang et al., 2018b; Zhou, 2020).

### Study characteristics

**FIG. 2.**
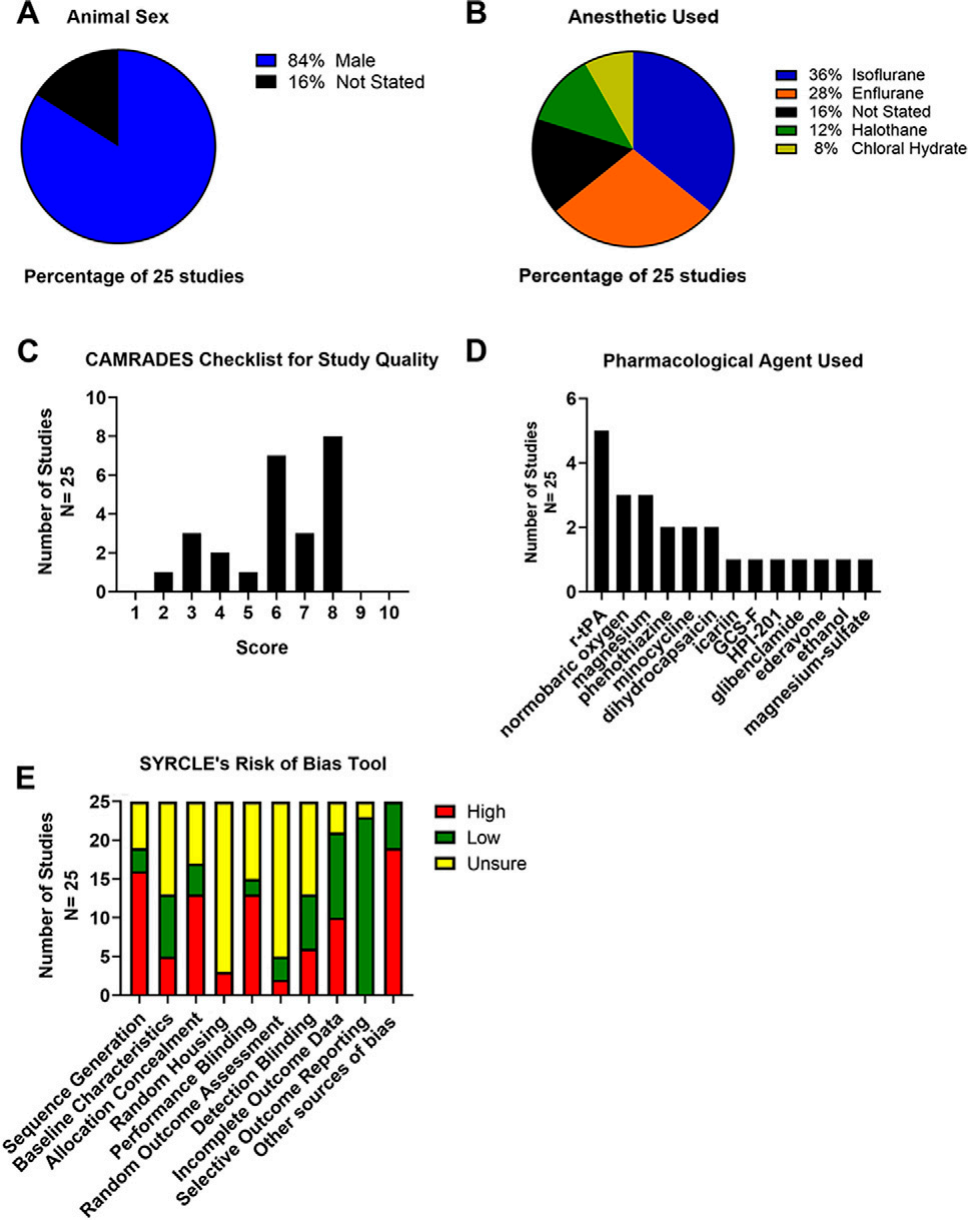
Analysis of experimental characteristics. **(A)** Percentages of male and female animals. No studies stated that they used female animals. **(B)** Breakdown of the different anesthetics used. **(C)** An evaluation of studies’ methodological quality using the CAMARADES Checklist for Study Quality. The median methodological quality score was 6, indicating moderate study quality. The range of scores was from 2 to 8. **(D)** Summary of SYRCLE’s risk of bias tool. **(E)** A pie chart illustrating the pharmacological agents used for cotherapy with TH. There were five studies that evaluated the combination of recombinant tissue plasminogen activator (r-tPA) and TH, with only—one to three studies for all of the other combinations. GCS-F, granulocyte colony-stimulating factor; TH, therapeutic hypothermia.

All studies used rodents—84% of which used males whereas 16% did not state the sexes used (Fig. 2A). Only one study (4%) used hypertensive animals, and no studies looked at other comorbidities. Thirty-six percent of studies used isoflurane as an anesthetic (Fig. 2B). Sixty-eight percent of studies used the middle cerebral artery occlusion model. No studies were preregistered. Sixteen percent of studies conducted *a priori* sample size calculations. Finally, 20% used 8- to 13-week-old animals, 24% used “adult” animals, and the remainder did not state ages. As such, it appears that no studies used aged animals.

### Methodological quality and certainty analysis

Briefly, 84% of studies reported randomization, 60% stated blinded assessment of outcome, and 28% reported blinded induction of ischemia (Supplementary Table S1). The median methodological quality score was 6, indicating moderate study quality (Fig. 2C). Inter-rater reliability was high (Cohen’s kappa = 1.0).

See Supplementary Table S2 for a detailed view of our SRoB assessment. Overall, selective outcome reporting had the lowest risk of bias among the other categories (Fig. 2D). Inter-rater reliability was high (Cohen’s kappa = 0.95).

Based on the CAMARADES checklist and the GRADE guidelines, initial certainty was “high,” as most of the studies adhered to randomization (Balshem et al., 2011; Hooijmans et al., 2018). Risk of bias was serious because of methodological limitations displayed in the SRoB results. Inconsistency and publication bias were serious because of outcomes having moderate-to-high heterogeneity and asymmetrical funnel plots, respectively. Finally, indirectness and imprecision were not serious because of studies addressing the PICOS outline and having narrow confidence intervals, respectively. Thus, the overall certainty of the evidence for our findings was low.

### Characteristics of TH

Seventy-six percent of studies used an external whole-body method of cooling (i.e., systemic cooling). Twelve percent used an external local method of cooling (i.e., only a specific part of the brain was cooled). Finally, 8% used an internal local method of cooling, and 4% did not state their cooling method.

Target temperatures ranged from 32°C to 35°C, with all studies regulating body temperature during surgery and TH administration. Twelve percent of studies monitored brain temperature. Cooling maintenance ranged from 1 hour to 48 hours, with the median being 3 hours. Sixty percent of studies did not state rewarming rate, 24% rewarmed for ≥1 hour, 8% for <1 hour, and 8% either spontaneously or gradually rewarmed. The induction period of TH ranged from 1 hour to 10 hours after stroke onset. Finally, 76% of studies explicitly stated their justification for their TH parameters, whereas 24% of studies did not provide any explanation.

### Characteristics of pharmacological agents

See Figure 2E and Supplementary Table S3 for an overview of all the data extracted for the agents (e.g., mechanism of action). Thirty-six percent of studies used multiple doses, and 64% used single dose. Dosing frequency varied, with the most common being only once after stroke onset. Twenty-four percent of studies gave a loading dose followed by continuous administration of the drug over several minutes or hours, whereas 16% administered treatment once daily for several days. Finally, 72% of studies explicitly justified (e.g., providing commentary and references) their dosages, and 28% of studies did not provide any explanation.

### Characteristics of combination treatments

Fifty-two percent of studies maintained their combination treatments until TH stopped, 40% until the drug stopped, and 8% did not state the duration. Finally, 76% gave both drug and TH simultaneously, 20% staggered the administration, and 4% did not state when they began the combination treatment.

### Infarct volume results

**FIG. 3.**
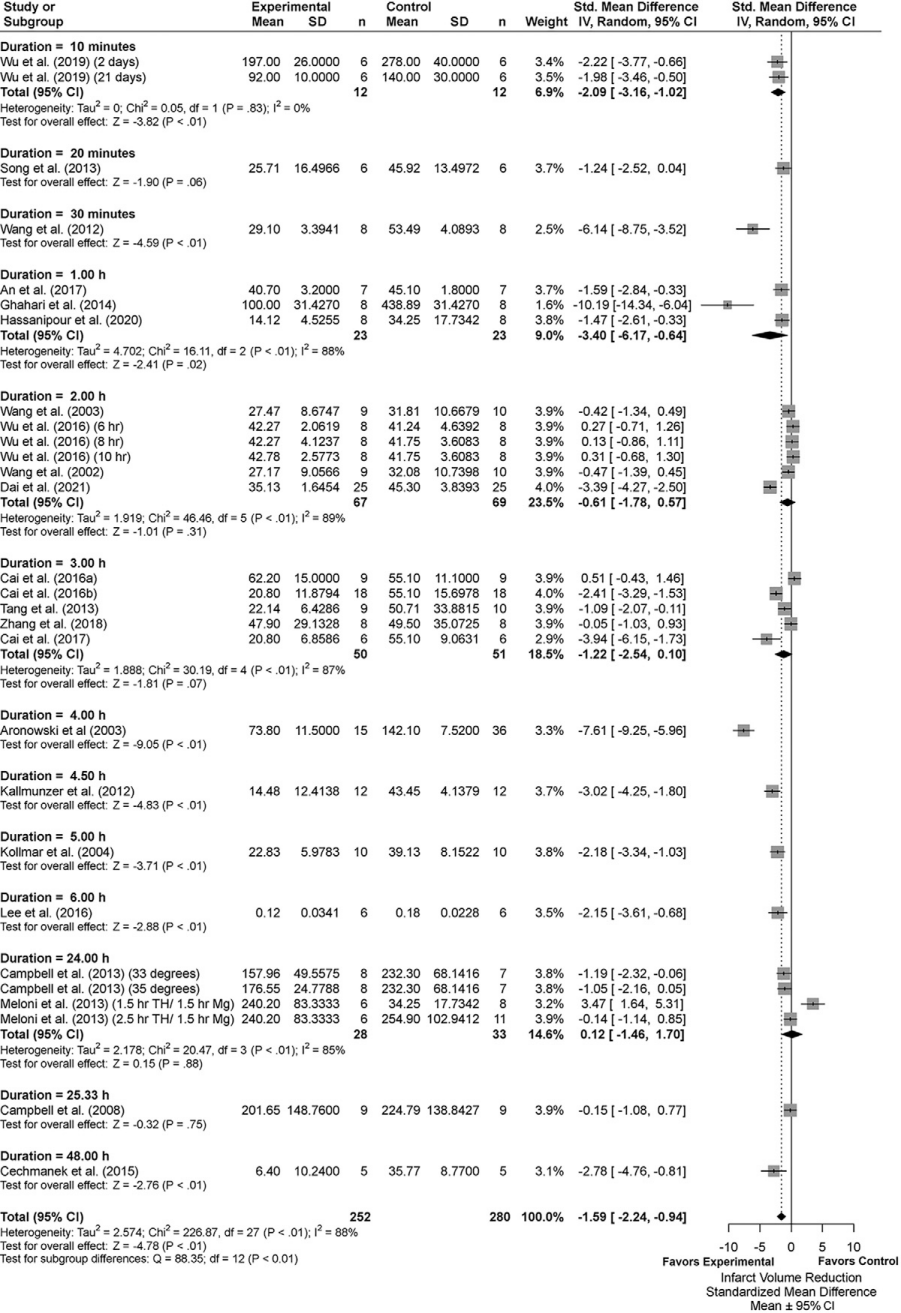
Forest plot of the TH versus NORMO group comparisons of studies that investigated infarct volume (standardized mean difference ± 95% CI). Subgroup analysis showed that a TH duration of 4 hours significantly reduced infarct volume the most. Overall effect size estimates were heterogenous, likely because of study design differences. CI, confidence interval; TH, therapeutic hypothermia.

**FIG. 4.**
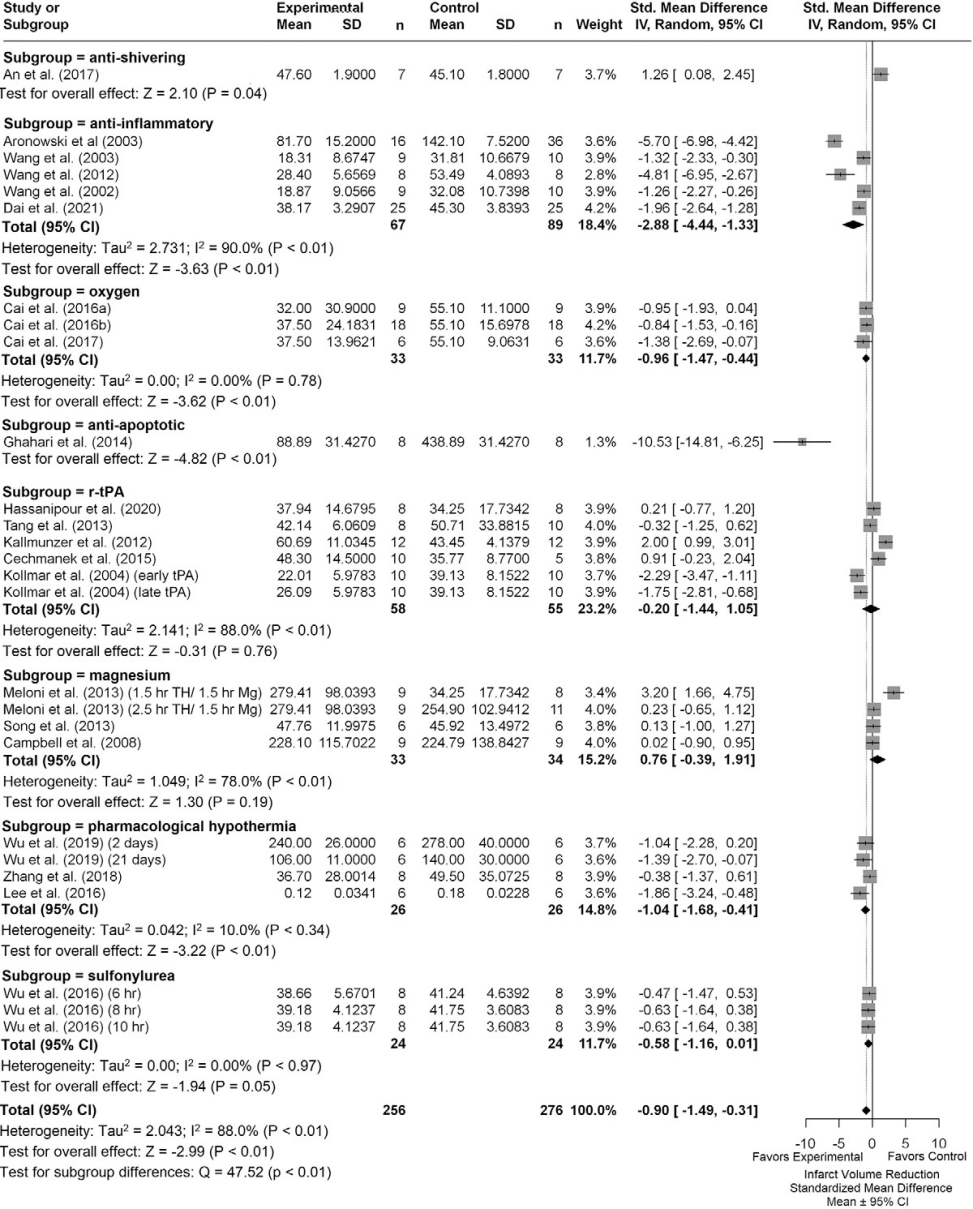
Forest plot of the DRUG versus NORMO group comparisons of studies that investigated infarct volume (standardized mean difference ± 95% CI). Subgroup analysis showed that antiapoptotic agents reduced infarct volume the most. Overall, huge heterogeneity was observed. CI, confidence interval.

We analyzed a total of 31 comparisons as some studies assessed multiple TH durations. Eighteen studies determined % infarction, 2 used infarct volume ratio, 1 used reduced infarct volume (% reduction compared with control), and 2 reported total infarct volume in cubic millimeters. The latest poststroke infarct volume assessment was on day 21. Supplementary Table S4 provides descriptive characteristics of infarct volume assessments for each group comparison (e.g., TH+DRUG vs. TH). Briefly, the summary statistics for our major group comparisons are as follows: TH versus NORMO (SMD: −1.59, CI: [−2.24, −0.94]; Fig. 3); DRUG vs. NORMO (SMD: −0.90, CI: [−1.49, −0.31]; Fig. 4); TH+DRUG versus TH (SMD: −0.96, CI: [−1.50, −0.43]); and TH+DRUG versus DRUG (SMD: −1.42, CI: [−1.98, −0.86]). The TH+DRUG versus DRUG and TH versus NORMO group revealed an additive effect as the CIs overlapped with one another. Sensitivity analyses also indicated an additive effect (TH+DRUG vs. DRUG SMD: −1.07, CI: [−1.48, −0.57]; TH vs. NORMO SMD: −1.09, CI: [−1.62, −0.55]; TH+DRUG vs. TH (SMD: −0.64, CI: [−1.06, −0.23]).

Subgroup analysis of the TH vs, NORMO groups revealed that the 4-hour duration produced the most beneficial effects regarding infarct volume reduction (SMD: −7.61, CI: [−9.25, −5.96], *p* < 0.01, Fig. 3). Removing studies with high statistical leverage (i.e., those having effects 3 times larger than the overall meta-analytic effect) caused the 4.5-hour subgroup to provide the greatest reduction of infarct volume (SMD: −3.02, CI: [−4.25, −1.80], *p* < 0.01). Overall heterogeneity was high (*I*^2^ = 88%, *p* < 0.01). Trim-and-fill analysis and Egger regression indicated missing negative or null data (*p* = 0.0119; Supplementary Fig. S1A).

Subgroup analysis of the DRUG versus NORMO groups by agent categories indicated that antiapoptotic drugs provided the largest infarct volume reduction (SMD: −10.53, CI: [−14.81, −6.25], *p* < 0.01; Fig. 4). Removing studies with high statistical leverage caused anti-inflammatories to provide the greatest effect in reducing infarct volume (SMD: −1.64, CI: [−2.13, −1.15], *p* < 0.01). Trim-and-fill analysis and Egger regression did not reveal any major publication bias (*p* = 0.2437; Supplementary Fig. S1B).

To investigate the effects of various parameters on the combination treatments, a meta-regression was performed on the TH+DRUG versus NORMO group using agent categories as predictors. Magnesium with TH provided the greatest infarct volume reduction (SMD = −11.63; *n* = 7) even after sensitivity analyses were conducted (SMD = −2.69).

### Behavioral results

**FIG. 5.**
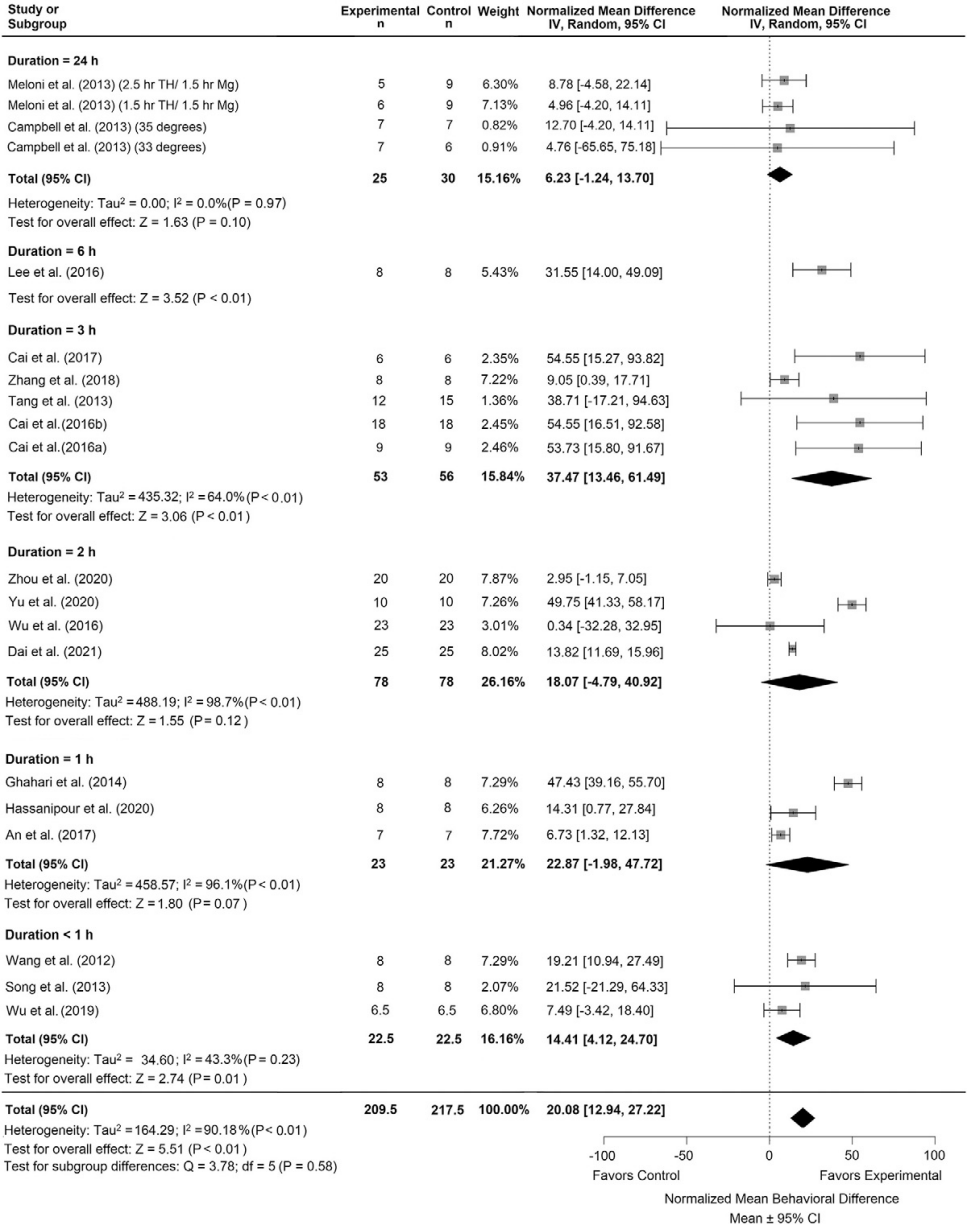
Forest plot of the TH versus NORMO group comparisons of studies that investigated behavior (normalized mean difference ± 95% CI). Subgroup analysis showed that a TH duration of 3 hours had the greatest effect on behavior outcomes compared with other timepoints. Overall heterogeneity was lower compared to the comparison for infarct volume. CI, confidence interval; TH, therapeutic hypothermia.

**FIG. 6.**
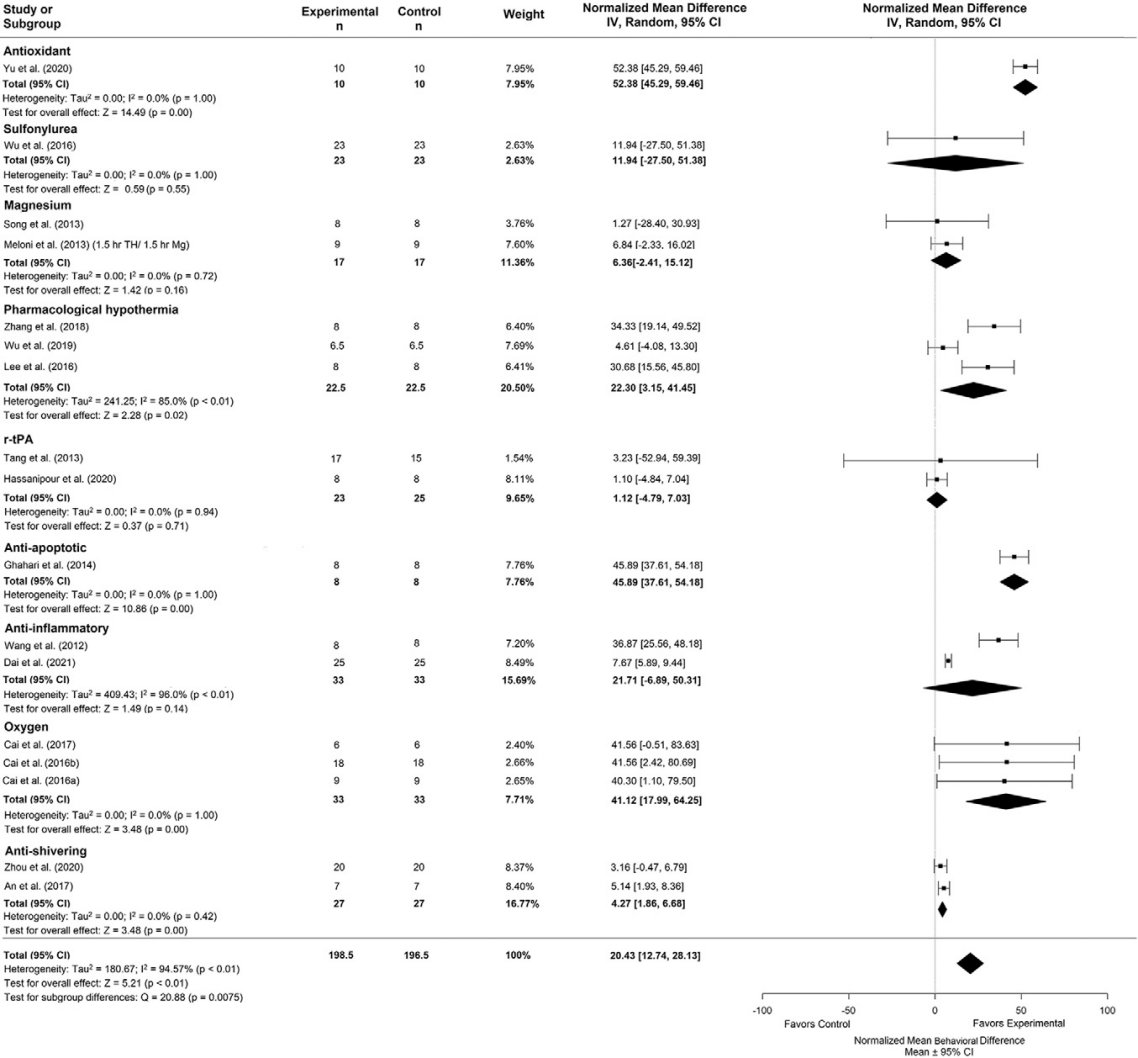
Forest plot of the DRUG versus NORMO group comparisons of studies that investigated behavior (normalized mean difference ± 95% CI). Subgroup analysis showed that antioxidants had the greatest effect on behavior outcomes compared to other timepoints. CI, confidence interval.

Twenty-one comparisons were made across 18 studies assessing TH duration. The latest poststroke behavioral assessment was on day 28. If studies used multiple tests, we statistically combined them (Vesterinen et al., 2014). Descriptive characteristics of the behavioral results can be found in Supplementary Table S5. Briefly, the summary statistics for our major group comparisons are as follows: TH versus NORMO (NMD: 20.08%, CI: [12.94, 27.22]; Fig. 5); DRUG versus NORMO (NMD: 20.43%, CI: [12.74, 28.13]; Fig. 6); TH+DRUG versus TH (NMD: 23.25%, CI: [6.97, 39.53]; sensitivity analysis: NMD: 19.72%, CI: [12.37, 27.07]); TH+DRUG versus DRUG (NMD: 19.83%, CI: [13.89, 25.78]). The TH+DRUG versus DRUG and the TH versus NORMO group revealed an additive effect of the agent with TH.

Subgroup analysis of the TH duration times for the TH versus NORMO comparison indicated that the 3-hour duration improved behavior the most compared with other timepoints (NMD: 37.47%, CI: [13.46, 61.49], *p* < 0.01, Fig. 5). Trim-and-fill analysis and Egger regression (*p* = 0.1742) did not reveal any major publication bias (Supplementary Fig. S1C).

Subgroup analysis of the agent categories for the DRUG versus NORMO comparison revealed that antioxidants provided the greatest improvement on behavior outcome (NMD: 52.38%, CI: [45.29, 59.46], *p* < 0.001; Fig. 6). Trim-and-fill analysis and Egger regression (*p* = 0.28) did not reveal any publication bias (Supplementary Fig. S1D).

Meta-regression using the TH+DRUG versus NORMO group revealed that magnesium provided the greatest impact on improving behavior when combined with TH (NMD = 79.10%; *n* = 6).

### Edema volume results

**FIG. 7.**
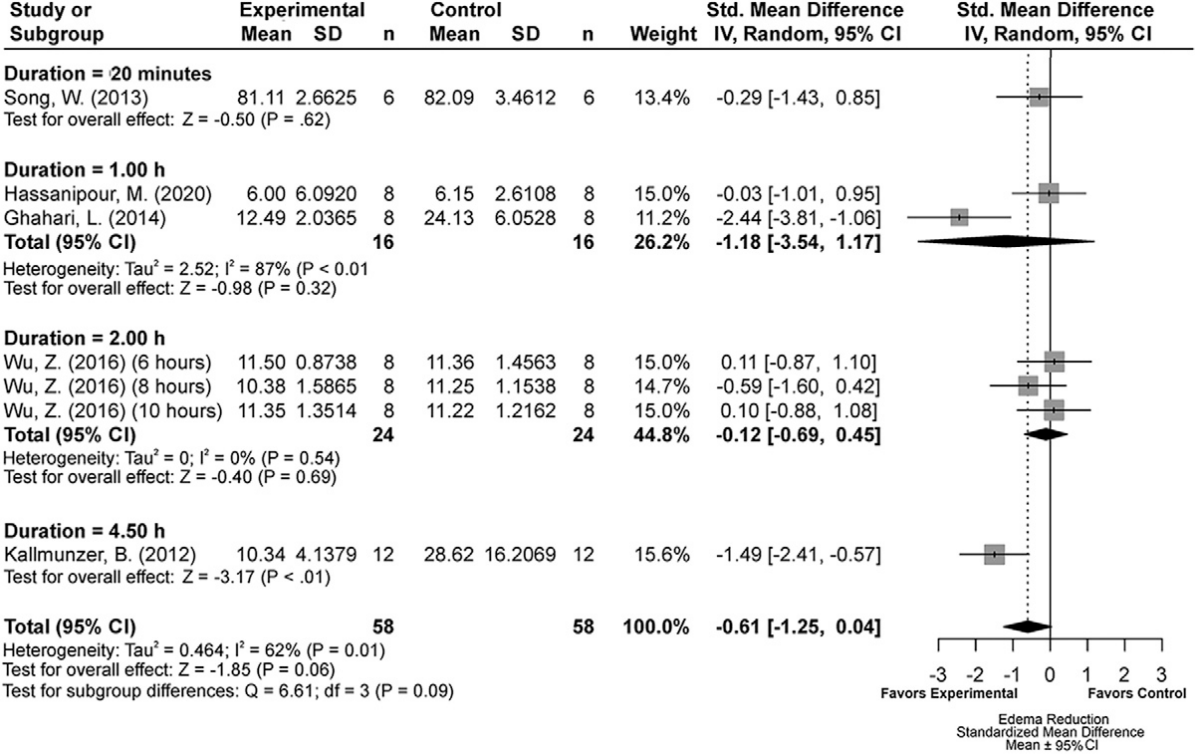
Forest plot of the TH versus NORMO group comparisons of studies that investigated edema volume (normalized mean difference ± 95% CI). Subgroup analysis showed that a 4.5-hour TH duration produced the most beneficial effects regarding edema volume reduction. CI, confidence interval; TH, therapeutic hypothermia.

**FIG. 8.**
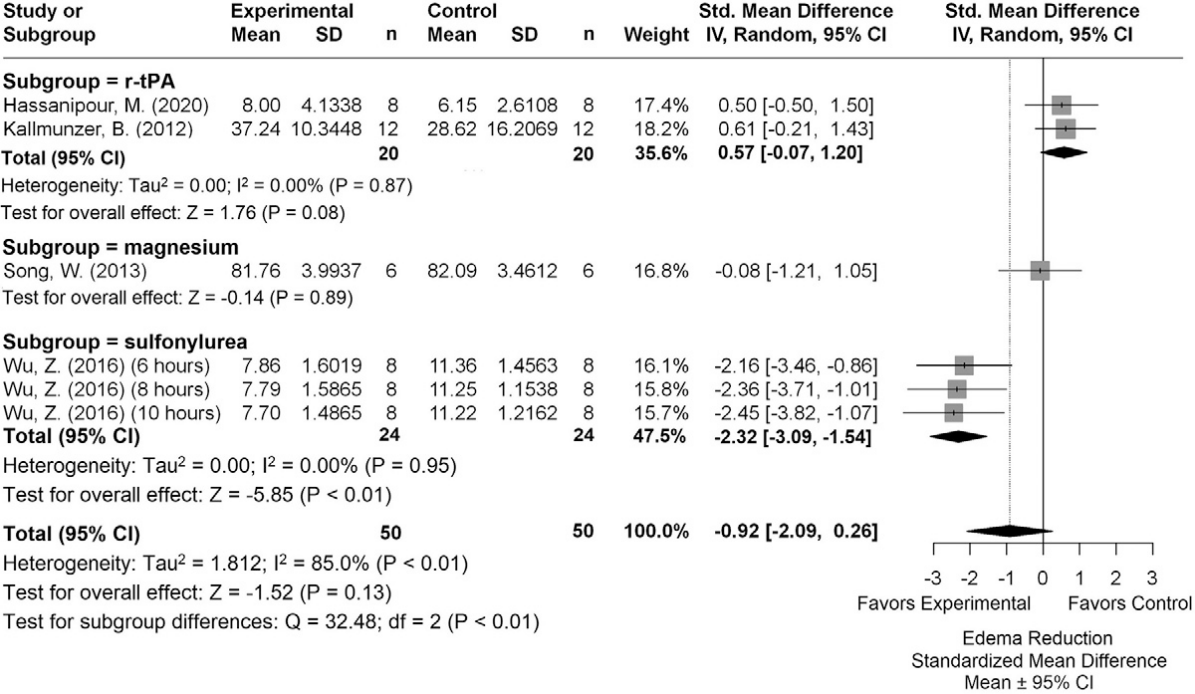
Forest plot of the DRUG versus NORMO group comparisons of studies that investigated edema volume (normalized mean difference ± 95% CI). Subgroup analysis showed that sulfonylureas produced the most beneficial effects regarding edema volume. CI, confidence interval.

We analyzed 6 experiments that evaluated edema because of one study having multiple TH durations. Of these, 1 used histological methods, and 5 used the tissue wet weight-dry weight method (Supplementary Tables S1 and S6). Briefly, the summary statistics for our major group comparisons are as follows: TH versus NORMO (SMD: −0.61, CI: [−1.25, 0.04]; Fig. 7); DRUG versus NORMO (SMD: −0.92, CI: [−2.09, 0.26]; Fig. 8); TH+DRUG versus TH (SMD: −1.87, CI: [−3.63, −0.10]); and TH+DRUG versus DRUG (SMD: −1.19, CI: [−1.89, −0.49]). The TH+DRUG versus DRUG and TH versus NORMO comparisons revealed an additive effect of the agents with TH.

Subgroup analysis of the TH duration times for the TH versus NORMO comparison indicated that a 4.5-hour duration produced the biggest edema volume reduction (SMD: 1.49, CI: [−2.41, −0.57], *p* < 0.01; Fig. 7). Egger regression analysis (*p* = 0.47) and trim-and-fill analysis did not reveal major publication bias (Supplementary Fig. S1E).

Subgroup analysis of the DRUG versus NORMO comparison revealed that sulfonylureas influenced edema volume reduction the most (SMD: −2.32, CI: [−3.09, −1.54], *p* < 0.01; Fig. 8). Egger regression and trim-and-fill analysis indicated publication bias (*p* = 0.0062, Supplementary Fig. S1F).

Our meta-regression using the TH+DRUG versus NORMO group revealed that magnesium significantly impacted edema reduction when combined with TH (SMD = −2.09; *n* = 1).

## Discussion

Despite the abundance of preclinical research in ischemic stroke on the topic of TH, our meta-analysis is one of the few that have solely focused on combination therapies (O’Collins et al., 2012). We identified only 25 studies that tested delayed TH with a drug cotherapy. Most studies used healthy male rats and the same MCAO model. Only one to five studies were included within each agent category (median = 1). Out of the 14 agents combined with TH, only caffeinol was implemented into clinical trials, which was unsuccessful (Martin-Schild et al., 2009). Despite these limited data, our findings indicate that combining different sets of drugs with TH led to additive beneficial effects toward behavior, infarct volume, and cerebral edema following ischemic stroke. Longer TH durations (e.g., 3–4.5 hours) provided the biggest effect on outcomes. For the agent categories, sulfonylureas provided the biggest effect on edema (SMD = −2.32). The best outcomes for infarct volume (SMD = −1.64) and behavior (NMD = 52.38) were produced by anti-inflammatories and antioxidants, respectively. Our results suggest that magnesium may be the best agent to use alongside TH, at least in animal models of focal ischemia. Similarly, our results indicate additive effects of combination therapies, which bodes well for the use of these interventions. However, there are some limitations with respect to estimating efficacy because of the limited amount of data, wide CIs, and variations among endpoints and heterogeneities, indicating a lack of precision in estimating benefit. These limitations were also seen in the subgroup analyses for TH duration and agent categories. Wide CIs, likely because of modest sample sizes and model variability, provide less certainty in the conclusion (du Prel et al., 2009). In sum, our analysis reveals a small amount of literature with an insufficient range in testing. As such, there must be lower confidence in this literature regarding how well they predict the efficacy of drugs combined with TH.

Animal model characteristics were homogenous, although the actual experimental designs were not. Only one study used rats with hypertension, but they did not do a comparison study using rats with no comorbidities (Campbell et al., 2013). Effect sizes might also shrink when studies consider comorbidities (Schmidt‐Pogoda et al., 2020). Most studies also used young male animals, which may have likely overestimated effect sizes. Similarly, our analysis of Risk of Bias (RoB), based on the CAMARADES and SYRCLE tools demonstrated low certainty, high heterogeneity, and publication bias, which can also lead to overestimated effect sizes (Hooijmans et al., 2014). Future studies should use RoB tools and refer to ARRIVE and STAIR guidelines when reporting methods and results, as well as publish their research protocol to ensure that they are describing necessary information.

Our meta-regression model suggested that magnesium and TH had the greatest benefit in combination, compared with other agents, for all outcomes measured. This combination has also been tested in global ischemia models, and results indicate that TH and Mg^2+^ together greatly improved neuronal survival compared to the monotherapies (Zhu et al., 2005). Depending on the endpoints, between one and seven studies using Mg^2+^ were included in the meta-regression analyses, as some used multiple doses. Most studies administered the agent intravenously (IV) and at the same time as TH except Campbell et al. (2008), who administered magnesium 2 hours poststroke onset and TH 2, 4, or 6 hours poststroke onset. Our analyses only included their 2-hour postischemia administration, as the other two timepoints did not test the treatments individually, indicating insufficient controls used. None of the Mg^2+^ studies justified why they applied the treatments simultaneously, but it may be because of them wanting to maximize the efficacy of the combination therapy and potentially create a synergistic effect. Furthermore, all studies using Mg^2+^ administered the treatment within 3 hours after stroke onset, which was recommended in the Field Administration of Stroke Therapy–Magnesium trial (Saver et al., 2004). The administration route used reflects other preclinical and clinical stroke literature using Mg^2+^, with IV being deemed as a safe and reliable method (Saver and Starkman, 2011).

According to our categorization of synergistic and additive effects, magnesium can be classified as having additive effects when combined with TH for infarct volume, behavior, and edema volume. In general, the implication of Mg^2+^ being a potential cotherapy drug with TH is of importance, as Mg^2+^ levels in most stroke patients are low compared with healthy individuals (Turley et al., 2005). Magnesium is also an easy and safe drug to administer, on top of being relatively available compared with other drugs. However, magnesium did not provide any benefit when used alone. The timing of Mg^2+^ administration in the preclinical studies included was earlier compared with clinical trials suggesting benefit (Campbell et al., 2013; Meloni et al., 2013). Clinical trials have also obtained mixed results, with one study of 107 patients with acute ischemic stroke finding benefit after administering Mg^2+^ within 12 hours (Afshari et al., 2013) and recent Phase 3 studies with 1700 patients not finding significant functional outcome benefits (Saver et al., 2015). These recent trials indicate some concerns regarding the use of Mg^2+^ for ischemic stroke patients. For instance, optimal treatment parameters (e.g., dosage, timing) for Mg^2+^ may not be fully determined yet if Phase 3 trials are not successful. Other factors such as sample sizes and the clinical trial quality may also affect these negative results. Indeed, recently completed Mg^2+^ trials for ischemic stroke were in Phases 2 and 3, indicating our need to determine safety and efficacy parameters first before administering Mg^2+^ outside of clinical trials. As such, more research needs to be conducted to evaluate the use of Mg^2+^ clinically and with other cytoprotection methods, such as TH, as there are currently no clinical trials investigating this combination therapy. Similarly, the use of optimal protocols for each agent category and TH must be given further consideration. Although we presently found additive or synergistic effects, the same final level of benefit might arise with a single therapy if a better dosage was used. Given that very few authors conducted proper dose–response work (e.g., ideal treatment parameters), it is likely that most did not use optimal dosing TH protocols. Owing to a lack of data, we could not identify optimal TH parameters. The optimal TH duration for cytoprotection is still unknown, which may cause the efficacy of the treatment to differ between clinical and preclinical studies (Kuczynski et al., 2020; McCann and Lawrence, 2020). Many TH studies use a restricted range in cooling, depth, and rewarming rates; therefore, it may be the case that suboptimal cooling protocols were inevitably used. In our case, however, there was a very broad range in treatment parameters, which indicates that authors may not have based their justification on the same literature or used comparable reasoning. Regarding cooling duration, a recent analysis demonstrated a positive association between duration and efficacy (Eberle et al., 2023). In addition, our subgroup analyses on TH duration revealed that majority of studies used shorter durations (<3 hours) even after sensitivity analyses were conducted. Studies generally used a limited temperature range (31–35°C), and only a few justified why they administered certain temperatures over others. A large percentage also did not state important TH parameters (e.g., rewarming rate). These poor study design qualities in preclinical studies led to experimental bias and hindered translation effort (Huang et al., 2019). Future work must fully report their methodologies to prevent biases from occurring. Finally, most studies allowed for reperfusion in their ischemic stroke models, which mimics the use of tissue plasminogen activator or MT; however, no comparisons were made to determine the effects of reperfusion versus no reperfusion in affecting the combined efficacy of TH and/or DRUG in these studies.

Subgroup analysis on agent categories suggested that antiapoptotic drugs originally had the biggest effects on all outcomes. After sensitivity analyses were conducted because of an outlier (Ghahari et al., 2014), anti-inflammatories provided the most benefit in reducing infarct volume, whereas sulfonylurea drugs had the biggest effect on edema volume and behavior. Large heterogeneity was seen for the antiapoptotic and anti-inflammatory groups, indicating study differences. However, this was based upon a relatively small number of studies, and most had modest sample sizes (*n* < 12). No studies also conducted dose–response analyses. Similar to our results, previous meta-analyses on combination therapies also report substantial heterogeneity and potential publication bias, resulting in constrained conclusions (O’Collins et al., 2012). In addition, the largest apparent effect size was with an antiapoptotic agent (granulocyte colony-stimulating factor) (Ghahari et al., 2014). The SMD was also quite large with this study as variability was remarkably small. As well, the behavioral data were analyzed in an unconventional way in apparent violation of assumptions of the analysis of variance, which they used. Overall, we must urge caution in interpreting the drug only comparisons.

### Limitations

In addition to the issues with the literature we analyzed, there are several limitations with our review. First, it may be possible that we missed important literature, such as those that were not published in English. Furthermore, we limited our studies to delayed therapies (TH delay of ≥ 1 hour after stroke onset). We limited our treatment administration after stroke onset to have higher face validity (possible treatment times in the clinic under optimal conditions). This undoubtedly limited our dataset. We also opted for a 1-hour delay exclusion cutoff, but it is possible that earlier treatment combinations would have yielded the same findings. The small number of studies included reflected the need for more consideration of future preclinical experiments focusing on paradigms that follow what is expected in clinical settings.

## Conclusion

Meta-analyses are important in scientific research as they provide an overview of the estimates of the effect of a specific treatment. Our results suggest that combination therapy with TH provided additive effects on all endpoints, which is, at least, proof of concept that combination therapies with TH will likely augment efficacy. Furthermore, we found significant differences between TH durations and agent categories when tested alone, although there are not enough data to confidently select the best therapy. More research is required using both TH and agents to establish the scope and magnitude of the treatment to inform its use in patients.

## Data Availability

The original data used in this study can be found in the Supplementary Materials, and further inquiries can be directed to the corresponding author.
