# Supplementary figures and images for: A Systematic Review and Meta-Analysis of Therapeutic Hypothermia and Pharmacological Cotherapies in Animal Models of Ischemic Stroke
Source: Ther Hypothermia Temp Manag. 2024 Dec 16;14(4):229–42. doi: 10.1089/ther.2024.0012 (PMC11685787; doi:10.1089/ther.2024.0012)

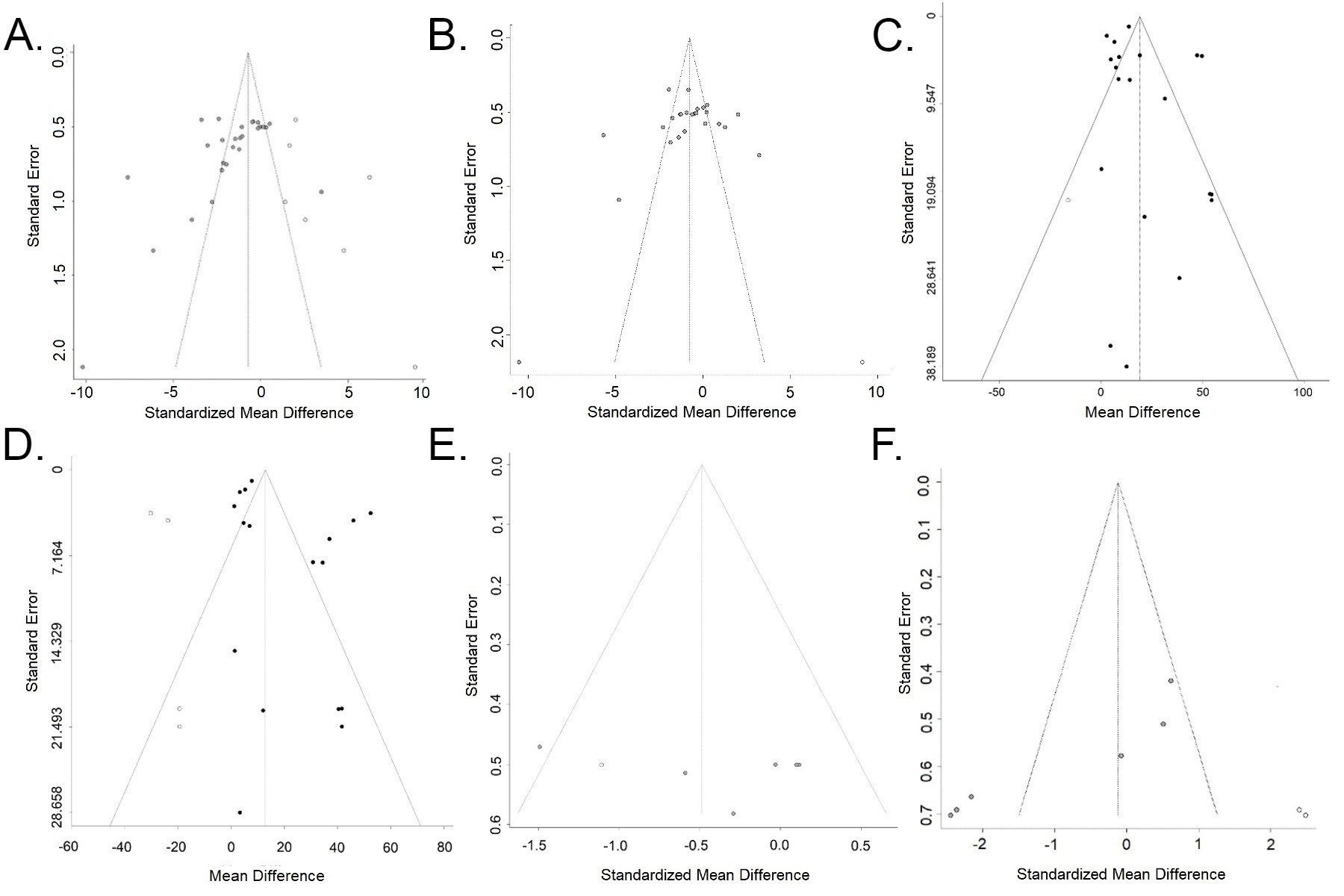

Supplement: Supplementary Figure S1 [file ther.2024.0012_figure_s1.jpg]
